# Complete loss of *SLC30A8* in humans improves glucose metabolism and beta cell function

**DOI:** 10.1007/s00125-025-06530-3

**Published:** 2025-09-29

**Authors:** Lindsey B. Lamarche, Christopher Koch, Shareef Khalid, Maleeha Zaman Khan, Richard Zessis, Matthew E. Clement, Daniel P. Denning, Allison B. Goldfine, Igor Splawski, Ali Abbasi, Jennifer L. Harrow, Christina Underwood, Kazuhisa Tsunoyama, Makoto Asaumi, Ikuyo Kou, Juan L. Rodriguez-Flores, Alan R. Shuldiner, Asif Rasheed, Muhammad Jahanzaib, Muhammad Rehan Mian, Muhammad Bilal Liaqat, Usman Abdulsalam, Riffat Sultana, Anjum Jalal, Muhammad Hamid Saeed, Shahid Abbas, Fazal Rehman Memon, Mohammad Ishaq, Allan M. Gurtan, John E. Dominy, Danish Saleheen

**Affiliations:** 1 Biomedical Research at Novartis, Cambridge, MA USA; 2 Columbia University Irving Medical Center, New York, NY USA; 3 Center for Non-Communicable Diseases, Karachi, Sindh Pakistan; 4 Present Address: Yarrow Biotechnology Inc., New York, NY USA; 5 Centre for Genomics Research, Discovery Sciences, AstraZeneca, Cambridge, UK; 6 Bioscience Metabolism, Research and Early Development, Cardiovascular, Renal and Metabolism (CVRM), Biopharmaceuticals R&D, AstraZeneca, Cambridge, UK; 7 Astellas Pharma Inc., Ibaraki, Japan; 8 Regeneron Genetics Center, LLC, Regeneron Pharmaceuticals, Inc., Tarrytown, NY USA; 9 Karachi Institute of Heart Diseases, Karachi, Sindh Pakistan; 10 Punjab Institute of Cardiology, Lahore, Punjab Pakistan; 11 Faisalabad Institute of Cardiology, Faisalabad, Punjab Pakistan; 12 Red Crescent Institute of Cardiology, Hyderabad, Sindh Pakistan

**Keywords:** Diabetes, Knockout, Pakistan Genome Resource, *SLC30A8*, South Asian, ZnT8

## Abstract

**Aims/hypothesis:**

Genetic association studies have demonstrated that partial loss of *SLC30A8* Function protects against type 2 diabetes in humans. We investigated the impact of complete loss of *SLC30A8* Function on type 2 diabetes risk and related phenotypes in humans.

**Methods:**

The Pakistan Genome Resource (PGR), a biobank comprising whole-exome and whole-genome sequences of 145,037 participants, was analysed for phenotypic associations with *SLC30A8* loss-of-function (LoF) variants. To follow up on the observations in the PGR, we conducted recall-by-genotype analyses of *SLC30A8* LoF heterozygotes and homozygotes, as well as their participating family members, using OGTTs.

**Results:**

We identified 18 *SLC30A8* knockouts, including homozygotes for a variant enriched in South Asians (Gln174Ter), and 1024 heterozygotes for LoF variants. Type 2 diabetes risk was lower in *SLC30A8* LoF heterozygotes and homozygotes relative to non-carriers, and the protective effect strengthens in a gene dose-dependent manner (OR_additive_=0.62; 95% CI 0.53, 0.72; *p*=1.1×10^–9^; OR_recessive_=0.34; 95% CI 0.12, 0.93; *p*=0.04). OGTTs in recall-by-genotype studies showed a gene dose-dependent reduction in glucose levels, coupled with elevated insulin.

**Conclusions/interpretation:**

The corrected insulin response, disposition index and insulin sensitivity index in LoF heterozygotes and homozygotes indicated higher glucose-stimulated insulin secretion with preserved beta cell function that was independent of BMI. These data suggest that therapeutic inhibition of *SLC30A8*, up to and including complete knockout, may treat type 2 diabetes safely and effectively.

**Graphical Abstract:**

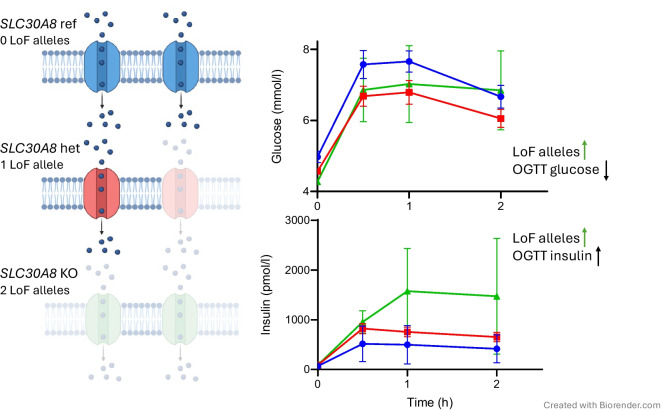

**Supplementary Information:**

The online version of this article (10.1007/s00125-025-06530-3) contains peer-reviewed but unedited supplementary material.


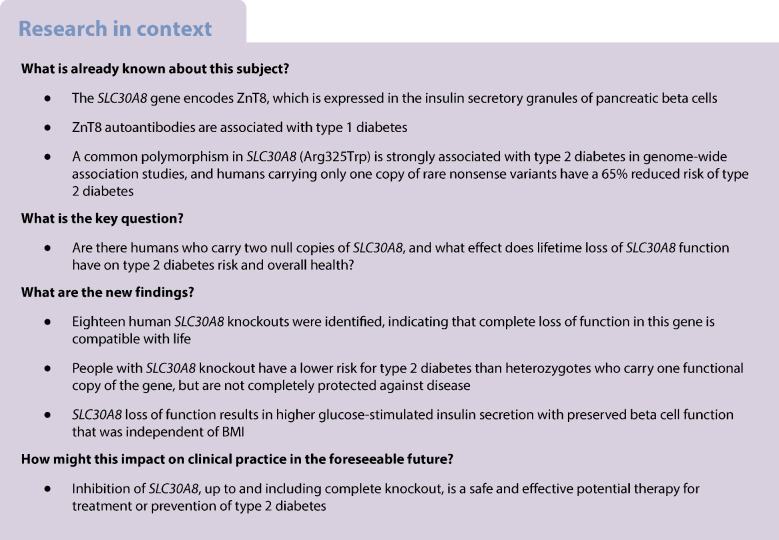


## Introduction

The *SLC30A8* gene encodes the zinc efflux transporter SLC30A8 (ZnT8) in pancreatic beta cells, and is strongly associated with type 2 diabetes in genome-wide association studies [1–10]. Common polymorphisms and rare heterozygous loss-of-function (LoF) mutations in *SLC30A8* are associated with reduced risk of developing type 2 diabetes, a disease that is characterised primarily by impaired insulin secretion and sensitivity. ZnT8 autoantibodies are associated with type 1 diabetes [11–13]. In vitro structure–function studies have proposed mechanisms by which *SLC30A8* LoF directly affects zinc-mediated insulin storage, processing and secretion [14–25], suggesting a possible path to type 2 diabetes treatment that is independent of weight loss. While weight loss therapies such as glucagon-like peptide-1 (GLP-1) agonists can be used to treat many patients with type 2 diabetes, identification of new targets may benefit individuals for whom weight loss drugs are contraindicated. Alternative therapies may also help patients who have been diagnosed with lean type 2 diabetes, for whom the benefit of weight loss therapies is not clear [26]. For these reasons, *SLC30A8* is a potential target in an underexplored pathway for therapeutic inhibition in diabetes.

Knockout mouse models have provided information regarding the effect of *Slc30a8* deletion on metabolic phenotypes such as fasting insulin, blood glucose levels, glucagon secretion and glucose-stimulated insulin secretion [27–34]. *Slc30a8* null mice consistently demonstrate lower zinc accumulation and atypical insulin granule formation, but show variable effects on insulin secretion and glucose homeostasis depending on the age, sex, diet and genetic background of the mouse [35–37]. However, *Slc30a8* null mouse phenotypes reproduce poorly in humans [38].

Genetic studies in participants of European descent identified two rare protein-truncating *SLC30A8* variants, Arg138Ter and Lys34SerfsTer50, for which heterozygosity was associated with 53% and 80% risk reduction for type 2 diabetes, respectively [8, 10]. Despite expansion of the recruitment effort to include approximately 150,000 individuals across multiple ancestry groups, no homozygotes of *SLC30A8* LoF variants were identified, limiting the scope of the study to partial loss of *SLC30A8* function. Questions remain about the effect and relative safety of complete loss of *SLC30A8* expression in humans.

South Asia is home to approximately two billion people who are disproportionately burdened by diseases such as type 2 diabetes and who are under-represented in most genetic studies [39]. Type 2 diabetes rates are high and increasing in South Asia, even for those with lower BMIs [39]. To build on previous human genetic studies of *SLC30A8* [8, 10], we used the Pakistan Genome Resource (PGR), a collection of nested case–control studies of Pakistani participants that has been assembled and is managed by the Center for Non-Communicable Diseases in Karachi, Pakistan. The PGR is a biobank that is enriched in participants from highly consanguineous families, leading to a higher prevalence of naturally occurring ‘human knockouts’, i.e. homozygotes for rare LoF variants, compared with other biobanks [40]. The PGR consists of 135,734 whole-exome sequences and 9303 whole-genome sequences, with patient medical history and numerous clinical measurements, including type 2 diabetes status, available for most participants. Using whole-exome sequencing, we identified and confirmed heterozygotes and homozygotes (‘knockouts’) for rare nonsense and damaging missense LoF variants in *SLC30A8*. For selected variants, we analysed in vitro protein expression to confirm LoF. We evaluated these variants for associations with phenotypic traits in human *SLC30A8* heterozygotes and knockouts identified through a family-based recall-by-genotype study.

## Methods

### Pakistan Genomic Resource overview

The PGR is a collection of nested case–control studies comprising 250,000 participants in Pakistan for whom extensive genetic, biomarker and lifestyle information has been collected, enabling evaluation of the genetic determinants of complex diseases and traits. Participants aged 18 years or older were enrolled from various hospitals and health units across Pakistan. Individuals meeting study-specific disease criteria were enrolled as case individuals, and age and gender-matched individuals from the same medical centres were enrolled as control individuals. Self-reported ethnicity was recorded for the majority of participants. No analyses made use of this information. Genetic principal components are used to control for population stratification. The institutional review board at the Center of Non-Communicable Disease (registration IRB 00007048) approved the study. All participants provided informed consent. Detailed methodology for the PGR has been reported previously [40, 41].

### Phenotype definitions

Type 2 diabetes was defined as individuals with an HbA_1c _≥48 mmol/mol (6.5%), those self-reporting diabetes or those using oral glucose-lowering drugs. Individuals with an age of onset of diabetes less than 30 years were excluded from the analysis. Control participants were defined as individuals with an HbA_1c_ <48 mmol/mol (6.5%), or individuals with no self-reported diabetes history or medication. Individuals with an elevated random blood glucose (above 8.32 mmol/l) were removed as control participants [41]. Type 2 diabetes patients and control participants were identified from the full set of PGR participants. eGFR was calculated using the CKD-EPI calculation [42]. The LDL-cholesterol analysis was limited to individuals who were not on cholesterol-lowering drugs, glucose levels were analysed for participants who were not on oral glucose-lowering drugs, and measurement of creatinine and eGFR was limited to participants without heart failure. Patients with myocardial infarction were enrolled at the time of the event as described previously [41]. Angina, atrial fibrillation/irregular heartbeats and hypertension were all self-reported. The control participants were healthy individuals without any cardiovascular disease history.

### Variant quality control (QC) and annotation

Exome sequencing was performed at two locations, the Broad Institute and the Regeneron Genetics Center, as described previously [40, 43]. All samples are sequenced at 30-fold coverage. Samples with low allele balance (<0.2) or low depth (<10) were set to missing. The male heterozygous call in the non-pseudoautosomal region of the X chromosome was set to missing. Variants that had a missing rate >5% were removed. High confidence-predicted LoF variants were annotated as stop gained, frameshift, splice donor or splice acceptor variants based on LOFTEE filtering. LoF variants only affecting the last exon or intron were removed. We used dbNSFP [44] to identify predicted damaging missense variants. Missense variants were categorised as ‘predicted damaging’ if they attained a REVEL score above 0.8, or if they were flagged as deleterious using five out of the six following predictors: FATHMM, Sift, PolyPhen, PolyPhen HDIV, LRT and Mutation Taster.

### Exome analysis

All quantitative traits were transformed using the rank-based inverse standard normal Function, applied within each genotyping batch. Quantitative traits were analysed using linear regression as implemented in regenie version 3.0 [45]. All analyses were adjusted for age, sex, age × sex, age^2^ and the top ten genetic principal components generated using common genotyping array SNPs. Gender was determined by self-reporting, with individuals excluded from the analysis if their self-reported gender was discrepant with computationally predicted genetic sex. For exome data, if genotyping array data was not available, principal components were derived from common exome SNPs (minor allele frequency >1%). Exome and genome data were analysed separately across sequencing centres and meta-analysed using inverse variance-weighted meta-analysis implemented in the latest version of METAL (https://csg.sph.umich.edu/abecasis/metal/download/). UK Biobank summary statistics were downloaded from GWAS catalogue study accession GCST90081710. Binary traits were analysed using a logistic regression model, with Firth fallback. Additive and recessive models were run by specifying the test parameter in regenie version 3.0 [45].

### Isolation of genomic DNA

DNA was extracted from leukocytes of peripheral whole blood using a reference salting-out method. DNA concentrations were determined by ultraviolet-based quantification with a Nanodrop 2000 spectrophotometer (Thermo Scientific).

### Sanger sequencing

Genomic DNA from whole blood collected from recalled participants was used to ascertain zygosity for variants of interest via Sanger sequencing. Sanger sequencing was performed at Macrogen (South Korea) or in-house at the Center for Non-Communicable Diseases. PCR primers were designed covering a region of approximately 200–300 bases around the variant. For in-house Sanger sequencing, specific primers were designed to amplify the region of interest using Platinum Master Mix (Thermo Scientific). This amplified DNA product was cleaned up using ExoSAP-IT Express PCR Product Cleanup (Thermo Scientific). It was then used for cycle sequencing with BigDye Terminator version 3.1 following addition of BigDye XTerminator (Thermo Scientific) for clean-up, and run on an Applied Biosystems SeqStudio genetic analyser (Thermo Scientific). Manufacturers’ protocols were followed for all kits.

### Recall-by-genotype study

Participants who were heterozygous or homozygous for *SLC30A8* LoF variants were contacted by the Center for Non-Communicable Diseases. After obtaining consent from the proband and from the family members, questionnaires regarding past medical and family history were administered by trained research staff, in the local language. Physical measurements such as height, weight, hip and waist circumference were measured in standing position by using height and weight scales. BMI was calculated as kg/m^2^. The waist to hip ratio was determined using standing waist and hip measurements. If standing measures were not possible, lying waist and hip measurements were taken. Blood pressure and heart rate were recorded using Omron Healthcare M2 blood pressure monitors. Non-fasting blood samples were collected and analysed as described previously [40, 43]. A random urine sample was also collected from each participant. The samples were stored temporarily in dry ice in the field, transported to the central laboratory based at the Center for Non-Communicable Diseases and stored at −80°C. Measurements for total cholesterol, HDL-cholesterol, LDL-cholesterol, triglycerides, VLDL, alanine aminotransferase and aspartate aminotransferase and creatinine were performed on serum samples using enzymatic assays, whereas levels of HbA_1c_ were measured using a turbidimetric assay in whole-blood samples (Roche Diagnostics). Urinary microalbumin was also measured using the urine sample.

### OGTT

Consenting participants were requested to adhere to a high-carbohydrate diet for 3 days prior to testing. Blood samples from participants who had fasted for 8 h were obtained. Participants were then administered glucose (75 g), orally and 3 ml blood samples were collected at 0, 30, 60 and 120 min. For each sample, plasma and serum were separated, stored on dry ice and transferred to a central location and stored at −80°C before measuring glucose levels for each time point. The corrected insulin response (CIR30) and Matsuda insulin sensitivity index (ISI) were calculated from fasting glucose and insulin measurements as previously described [46], and the disposition index (DI) was calculated as their product (CIR30 × ISI). Analyses were restricted to individuals meeting the following criteria: no reported history of diabetes, HbA_1c_ <48 mmol/mol (6.5%), and no reported use of glucose-lowering medication. Statistical tests were performed using an additive linear mixed model with age, BMI and self-reported gender as fixed effects, and family ID as a random effect. All analyses were performed using the Python module statsmodels (version 0.13.0) [47].

### In vitro expression and western blot

Human codon-optimised cDNAs corresponding to wild-type ZnT8B (NCBI reference NP_001166286) and missense and nonsense variants were cloned into the pcDNA3.1(+) mammalian expression vector with an N-terminal FLAG tag (DYKDDDDK) between the start methionine and the second amino acid residue. Transient transfection of adherent HEK293 cells in six-well plate format was performed using Lipofectamine 2000 (Invitrogen) according to the manufacturer’s instructions. Transfection optimisation for selected variants was performed by holding constant the total DNA:lipofectamine mass ratio at 1:3, and diluting the plasmid into empty vector such that the total plasmid content of each transfection mixture remained fixed at 2 µg but only 2, 1, 0.5, or 0.1 µg *SLC30A8* DNA was added per well. Cells were harvested 48 h post-transfection by enzymatic dissociation, washed in PBS, and pelleted by centrifugation at 500 *g* for 5 min. Pellets were resuspended in RIPA buffer supplemented with Halt protease inhibitor cocktail (Thermo Scientific), incubated for 30 min at 4°C with end-over-end rotation, and centrifuged at 16,000 *g* for 15 min to yield solubilised lysate. Lysate samples were analysed by SDS–PAGE and western blotting against the FLAG epitope. Detection was performed using FLAG M2 primary antibody (F1804, Sigma), HRP-conjugated anti-mouse IgG secondary antibody (HAF007, R&D Systems) and SuperSignal West Femto substrate (Thermo Scientific). Imaging was performed on an Amersham Imager 680 (GE Life Sciences). Transfections and western blots were performed in triplicate.

### Immunoassays

Circulating levels of insulin, GLP-1 (total), gastric inhibitory polypeptide (GIP) (total), C-peptide and proinsulin were evaluated using multiplexed U-Plex Metabolic Group 1 assay plates (catalogue number K151ACL-2, Meso Scale Discovery [MSD]) according to the manufacturer instructions. Briefly, 96-well multiplex plates were coated at 50 μl per well with biotinylated antibodies coupled to site-specific U-Plex linkers, allowing each analyte to self-assemble on unique spots in each well. Plates were incubated overnight at 4°C with shaking at 500 rev/min. Each well was washed three times with 150 µl wash buffer (provided in the kit) before addition of 50 µl recombinant protein or human plasma diluted in metabolic assay working solution (provided in the kit). Plasma was diluted eightfold for measurements of GIP, GLP-1, C-peptide and insulin, or fourfold for proinsulin measurements. After shaking at 500 rev/min for 2 h at room temperature, plates were washed three times with 150 µl wash buffer, treated with 50 µl of detection antibodies conjugated to electrochemiluminescent SULFO-TAG labels per well, and incubated at room temperature for 1 h with shaking. After three washes with wash buffer, 150 µl MSD-GOLD read buffer B was added to each well, and plates were imaged on a Sector S 600 MM imager (Meso Scale Discovery). The lower limits of quantification (LLOQ) for each analyte were in line with kit specifications (insulin 1.25 pmol/l; proinsulin 1.97 pmol/l; C-peptide 0.04 nmol/l; GIP total 3 pmol/l; GLP-1 total 54.3 pg/ml).

## Results

### *SLC30A8* is the strongest genetic signal for protection against type 2 diabetes across PGR and the UK Biobank

**Fig. 1:**
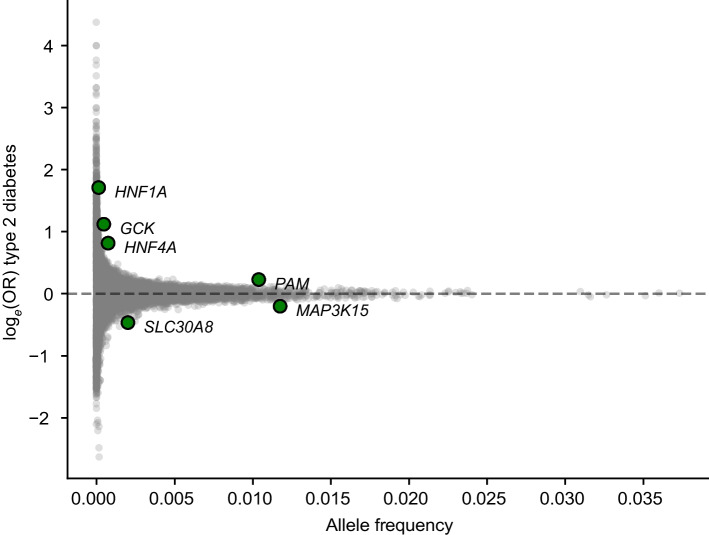
LoF and damaging missense gene burden exome-wide association studies for type 2 diabetes identified genes associated with susceptibility to or protection from type 2 diabetes. Significant hits (*p*<5×10^–7^) are shown in green. Six genes (*HNF1A*, *GCK*, *HNF4A*, *PAM*, *MAP3K15* and *SLC30A8*) were identified at exome-wide significance. Of the two genes for which LoF was associated with a lower risk of type 2 diabetes (*SLC30A8* and *MAP3K15*), the greatest reduction in risk was observed for *SLC30A8*

Genetic associations with type 2 diabetes in the PGR were identified by exome-wide association studies. To compare the effect of *SLC30A8* LoF with that of other genes, we performed an exome-wide gene burden test combining predicted LoF variants or LoF + damaging missense variants with allele frequency below 1%. To boost power, we performed a meta-analysis combining the PGR results with the UK Biobank gene burden summary statistics, in which similar gene burden definitions were used [48]. In total, our meta-analysis consisted of 44,162 type 2 diabetes patients and 392,238 control participants. A total of six exome-wide (*p*<5×10^–7^) significant hits were identified, including *SLC30A8* (*p*=5×10^−10^, OR=0.63; 95% CI 0.55, 0.73), with consistent effect size across the UK Biobank and PGR (*p*_heterogeneity_=0.83). Of these six genes, *SLC30A8* LoF was associated with the largest reduction in type 2 diabetes risk (Fig. 1). The only other protective hit for LoF variants reaching exome-wide significance was *MAP3K15*, which has been reported previously [49]. Summary statistics for the PGR and UK Biobank exome-wide association studies and meta-analysis are available in the electronic supplementary material (ESM Table 1).

### *SLC30A8* knockouts in the PGR have significantly lower risk for type 2 diabetes

Across 145,037 sequenced individuals, we identified 18 putative LoF variants with a cumulative allele frequency of 0.3% (ESM Table 2). These included 11 homozygous individuals and three individuals who were heterozygous for two different nonsense variants (Table 1). These variants were predicted to be *in trans* [50], hence these individuals were also included as *SLC30A8* knockouts. Gene burden analysis of LoF heterozygotes and homozygotes showed a 34% per allele lower type 2 diabetes risk (OR_additive_=0.66; 95% CI 0.56, 0.77; *p*=3×10^–7^).

**Table 1:** Clinical phenotypes of *SLC30A8* knockouts in the PGR

Participant number	*SLC30A8* LoF variant	Age (years)	Gender	BMI (kg/m^2^)	Glucose (mmol/l)	T2D	Prior CVD history	Hypertension	Total cholesterol (mmol/l)	Triglycerides (mmol/l)
1	Gln174Ter	43	Male	26.9	5.1	No	No	No	4.4	8.0
2	Gln174Ter	55	Male	25.5	6.6	No	No	No	6.1	4.9
3	Gln174Ter	43	Female	28.6	4.7	No	No	No	4.1	1.8
4*	Gln174Ter	65	Male	35.6	6.1	Yes (42)	Stroke (65)	Yes	1.8	1.2
5*	Arg138Ter	55	Male	24.7	11.8	No	MI (55)	No	4.0	1.0
6	Arg138Ter	68	Male	24.8	5.5	No	CAD (68)	Yes	4.3	6.1
7	Gln174Ter	55	Male	Not determined	6.6	No	Stroke (55)	Yes	3.1	2.7
8	Arg138Ter	43	Male	26.5	8.0	No	Stroke (43)	Yes	6.1	1.5
9	Asp295ArgfsTer42	44	Male	28.3	3.8	No	No	No	2.1	1.8
10	Arg165His	57	Male	22.5	4.8	No	No	No	4.8	4.9
11	Splice acceptor	57	Female	30.5	4.3	No	No	No	3.2	1.5
12	Gln174Ter	42	Male	32.5	23.8	Yes (42)	No	Yes	2.9	3.5
13	Arg165His	45	Male	Not determined	5.5	No	No	No	4.9	1.8
14	Arg165His	34	Male	27.6	4.5	No	No	No	4.6	1.5
15	Arg138Ter and Arg165His	55	Male	34.3	5.9	No	No	No	5.4	2.4
16	Gln174Ter and Tyr284Ter	52	Male	26	5.4	No	No	Yes	3.3	1.0
17	Gln174Ter and Tyr284Ter	59	Male	12.5	4.6	No	No	No	4.3	1.8
18	Arg138Ter and Gln174Ter	75	Female	19.1	4.8	No	MI (75)	No	1.8	0.8

Glucose and cholesterol were measured from non-fasting blood samples. For clinical outcomes, age of onset is provided in parentheses. Asterisks indicate individuals who were contacted for the recall study and participated

CAD, coronary artery disease; MI, myocardial infarction; T2D, type 2 diabetes

In addition to high-confidence LoF variants, we selected six missense variants that were predicted to be damaging (see Methods) or that were in conserved positions for functional profiling in cell culture (ESM Fig. 1). Protein expression in a heterologous cell system was used to evaluate potentially damaging missense variants. Five out of six variants showed substantially lower expression of monomeric SLC30A8 and higher levels of aggregated protein, and were categorised as LoF missense variants. The Arg165His variant showed a statistically significant reduction in the OR for type 2 diabetes (OR_additive_=0.55; 95% CI 0.37, 0.81; *p*=0.003), while other individual associations were not statistically significant for most variants due to low allele frequency.

**Fig. 2:**
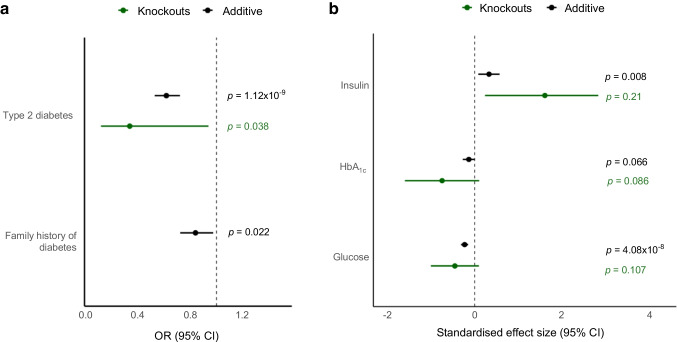
*SLC30A8* variants are associated with metabolic traits. (**a**) Association of LoF and damaging missense variants with type 2 diabetes and self-reported family history of diabetes. Additive and recessive models were run adjusting for age, age^2^, age × sex and the top ten genetic principal components, and whole-genome regression predictions were made using regenie. (**b**) Association with non-fasting glycaemic traits for LoF and damaging missense variants. Traits were rank inverse-normalised before analysis. Additive and recessive models were run adjusting for age, age^2^, age × sex and the top ten genetic principal components, and whole-genome regression predictions using regenie. Data points in (**b**) are β with 95% CI

We performed a second gene burden analysis that included the validated LoF missense variants (ESM Table 3). In this second analysis, three Arg165His homozygotes and one compound Arg165His/Arg138Ter heterozygote were included as *SLC30A8* knockouts (Table 1). Sixteen of the 18 knockouts did not have type 2 diabetes, representing a 66% decrease in type 2 diabetes risk (OR_recessive_=0.34; 95% CI 0.12, 0.93; *p*=0.04) relative to the reference population. The protective effect was also observed in heterozygotes, with a 37% decrease in type 2 diabetes risk (OR_additive_=0.62; 95% CI 0.53, 0.72; *p*=1.1×10^–9^), demonstrating a clear gene-dosage effect (Fig. 2a).

We also analysed the common variant Arg325Trp, which has been reported in previous studies to be associated with lower glucose and type 2 diabetes risk [4, 8, 9, 11, 13, 18, 21]. Arg325Trp was also associated with a significant decrease in type 2 diabetes risk (ESM Table 3); however, the effect size (OR=0.92; 0.90, 0.94; *p*=1.9 × 10^−15^) was much smaller compared with LoF variants. We observed similar results for glucose, insulin and HbA_1c_ (ESM Table 4). Adjusting the LoF gene burden analysis for the Arg325Trp genotype did not change the effect estimates, demonstrating that the LoF effect is independent of this variant (ESM Table 5).

### *SLC30A8* LoF is associated with lower random glucose and higher insulin levels in a gene dose–response pattern

To explore the effect of *SLC30A8* deficiency, we performed a burden test of LoF and damaging missense variants for association across a wide range of cardiometabolic and other closely related traits (ESM Table 6). We observed significantly lower random blood glucose (β=−0.22; 95% CI −0.31, −0.15; *p*=4.1×10^–8^) and higher insulin levels (β=0.37; 95% CI 0.09, 0.57; *p*=0.008) for *SLC30A8* LoF and missense variants with an additive model. HbA_1c_ levels also trended lower (β=−0.08; 95% CI −0.27, 0; *p*=0.07) (Fig. 2b). Non-fasted insulin levels were fivefold higher in knockouts (ESM Table 6). We also observed a nominal increase in C-peptide levels in knockouts only (*p*_recessive_=0.04). Self-reported family history of diabetes was also significantly lower for heterozygotes and knockouts in an additive model (OR_additive_=0.84; 0.72, 0.98; *p*=0.02). We observed nominal associations with increased risk of angina (self-reported) (*p*_additive_=0.002), higher heart rate (*p*_additive_=0.034), lower triglycerides (*p*_additive_=0.005), higher uric acid (*p*_additive_=0.003) and higher cystatin C (*p*_additive_=0.03). No significant associations were observed with other disease endpoints, such as myocardial infarction or other biomarkers, including liver enzymes aspartate aminotransferase (AST), alanine aminotransferase (ALT) and γ-glutamyltransferase (GGT).

### Recall-by-genotype and OGTT studies

*SLC30A8* LoF and Arg325Trp probands and their family members were recruited in the largest *SLC30A8* recall-by-genotype study to date. In total, 95 probands were recontacted, and 52 agreed to participate (54.7% success rate). For each recontacted participant and their consenting family members, *SLC30A8* genotype was confirmed through Sanger sequencing. Measurements of height, weight, heart rate, blood pressure and lipids were recorded. For this study, we recruited a total of 759 participants comprising 52 probands and 707 family members, including 132 LoF heterozygotes and three LoF homozygotes, as well as 317 heterozygotes and 89 homozygotes for the common variant Arg325Trp.

**Fig. 3:**
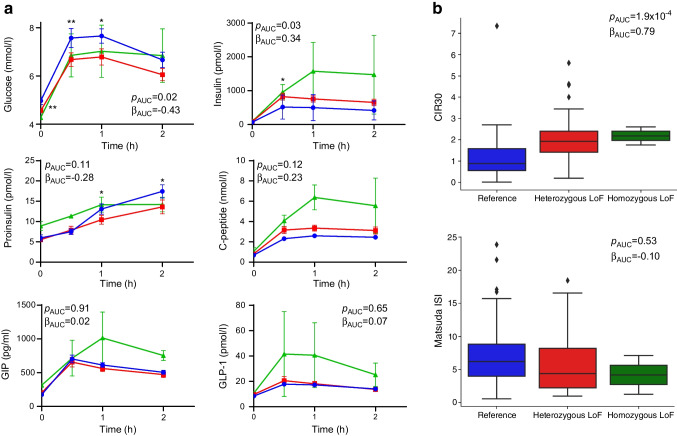
*SLC30A8* LoF carriers exhibit higher glucose tolerance and beta cell function compared with non-carriers. (**a**) OGTT samples from non-diabetic recall participants were analysed for plasma glucose, insulin, proinsulin, C-peptide, GIP and GLP-1. Reference individuals (*n*=80, blue) do not carry LoF alleles; heterozygous LoF individuals (*n*=42, red) carry one LoF allele. Of the homozygous LoF individuals (*n*=2, green), one carries two copies of Gln174Ter and the other is a compound heterozygote for two LoF alleles: Arg138Ter and Gln174Ter. Participants carrying Arg325Trp alleles were excluded. Analysis was performed using an additive linear mixed model, with age, BMI and self-reported gender as fixed effects, and family ID as a random effect. *p<0.05, **p<0.01. (**b**) The CIR30 and Matsuda ISI were calculated for each group from relevant insulin and glucose measurements. Centre line, median; box limits, upper and lower quartiles; whiskers, farthest data point within 1.5 × IQR; points, outliers

OGTT were performed on a total of 428 individuals, including 302 individuals from families carrying LoF variants. Serum samples from OGTT participants were analysed for the biomarkers glucose and insulin, as well as proinsulin, C-peptide and the incretins GIP and GLP-1 (Fig. 3a). In non-diabetic participants, we observed an apparent gene dose–response pattern in glucose levels, which were significantly lower for LoF heterozygotes and homozygotes in the fasted state (β=−0.56, *p*=9.5×10^–4^) and throughout the test (β=−0.43, *p*=0.02). We also observed higher insulin levels in LoF heterozygotes and homozygotes in the first 30 min of the test (β=0.59, *p*=2.6×10^–3^) and a nominally significant trend toward elevated insulin levels throughout (β=0.34, *p*=0.03). We also analysed OGTT results for the Arg325Trp variant (ESM Figs. 2 and 3). Compared with LoF heterozygotes and homozygotes, Arg325Trp heterozygotes and homozygotes showed similar patterns of lower glucose and higher insulin levels, but with a smaller effect size. For this comparison, individuals who carried both LoF and Arg325Trp alleles were excluded.

From collected OGTT data, the CIR30, Matsuda ISI and oral DI [46, 51] were calculated as measures of insulin sensitivity and beta cell function in non-diabetic LoF heterozygotes and homozygotes (Fig. 3b). A significantly higher CIR30 (β=0.79, *p*=1.9×10^–4^) was noted for LoF genotypes, but changes in the ISI did not reach statistical significance. The DI, calculated as the product of CIR30 and ISI, was also significantly higher for LoF genotypes (β=0.46, *p*=0.03).

## Discussion

Heterozygous loss of *SLC30A8* Function confers protection from type 2 diabetes [8–10]. Leveraging the high levels of rare variant homozygosity in the PGR, we identified the first complete human knockouts of *SLC30A8*, enabling characterisation of *SLC30A8* LoF in a gene dosage manner. Using a recall-by-genotype study design, we conducted phenotyping studies in the PGR for several metabolic parameters to understand the physiological mechanisms by which *SLC30A8* LoF protects against type 2 diabetes.

In the PGR, we identified 18 high-confidence LoF variants and eight damaging missense variants, of which 14 are specific to or enriched in South Asia. In vitro expression testing filtered computationally predicted damaging missense variants down to those that cause loss of expression, notably Arg165His. Although a limited method of assessing the Function of protein variants, heterologous expression testing supported the addition of carriers of damaging missense variants to our burden analysis to increase statistical power, without creating excess noise through inclusion of computationally predicted variants with little to no impact. In total, 18 homozygous and compound heterozygous individuals for *SLC30A8* LoF were identified, representing the first known human *SLC30A8* ‘knockouts’. These knockouts included both men and women with an age range of 43–75 years, many of whom have had children and grandchildren. Notably, the median age of the knockout group was higher than that of the heterozygote and reference group. These observations indicate that full *SLC30A8* LoF from birth is not only compatible with life, but generally well tolerated in humans.

We performed deep phenotyping studies on *SLC30A8* LoF heterozygotes and homozygotes in the largest recall-by-genotype study for *SLC30A8* to date, and the first to include human knockouts of *SLC30A8*. With a completion rate of 54.7%, this study represents a significant advance over recently reported recall frameworks for type 2 diabetes [52]. OGTTs were used as a minimally invasive means to measure glucose dynamics, beta cell function and insulin resistance. Three knockout patients were available for OGTTs; the first was a diabetic Arg138Ter homozygote, the second was a non-diabetic Gln174Ter homozygote, and the third was a non-diabetic Arg138Ter/Gln174Ter compound heterozygote. Loss of SLC30A8 Function was associated with gene dosage-dependent protection from type 2 diabetes. We also identified two *SLC30A8* knockouts with type 2 diabetes, indicating that protection from disease is not absolute, probably due to strong environmental factors such as diet or other polygenic risk factors that could not be evaluated in this study.

Separate recall and phenotyping efforts were conducted for rare LoF variants and the common protective variant Arg325Trp. We demonstrate that the measured phenotypes for Arg325Trp trend in the same direction as LoF (i.e. lower glucose and HbA_1c_, higher insulin), but the effect size in LoF carriers is larger and more likely to be statistically significant (ESM Table 4, ESM Figs 2 and 3). We also found that the Arg325Trp genotype does not significantly alter the results of LoF phenotype analysis (ESM Table 5).

In analysis of non-diabetic participants, glucose levels were significantly lower and post-load insulin levels were significantly higher in LoF heterozygotes compared with non-carriers, indicating higher glucose clearance. Among diabetic participants, the OGTT profiles of LoF heterozygotes and homozygotes were indistinguishable from those of non-carriers, suggesting that absence of *SLC30A8* is not associated with metabolic differences after onset of type 2 diabetes. From non-diabetic OGTT data, we calculated CIR30 and Matsuda ISI to quantify insulin response and sensitivity as a minimally invasive alternative to the traditional hyperglycaemic–euglycaemic clamp approach [46, 53]. LoF heterozygotes showed statistically significantly higher CIR30 values than non-carriers, suggesting that the higher glucose tolerance is due to enhanced insulin secretion. In contrast, no statistically significant changes were noted in the Matsuda ISI, suggesting that LoF heterozygotes did not develop insulin resistance. The oral DI, which approximates beta cell function for the degree of insulin resistance, was also higher in LoF heterozygotes compared with the control group. GIP and GLP-1 levels in the *SLC30A8* knockouts were not statistically significantly different relative to control participants, and were driven by one compound heterozygote with outlying results, while normal levels were observed in other heterozygotes and homozygotes. Among statistically significant phenotypes in heterozygotes and knockouts, are slightly elevated uric acid and cystatin C, both biomarkers for kidney function, as well as an elevated heart rate, all of which have previously been associated with elevated insulin [54–56].

The identification of *SLC30A8* knockouts is an important advancement over previous studies in which only heterozygotes were identified, allowing us to observe a gene dosage pattern for *SLC30A8* LoF for the first time. However, the statistical power of the analysis is still limited by the low number of homozygotes included in the study. More data from additional non-diabetic homozygotes is required to interpret observations such as the elevated incretins and to boost confidence in the degree of type 2 diabetes protection conferred by *SLC30A8* LoF. Altogether, these analyses support that *SLC30A8* LoF reduces type 2 diabetes risk through a mechanism of enhanced insulin secretion and improved overall beta cell function [9, 10], and that the magnitude of this effect may be proportional to the degree of *SLC30A8* LoF.

In summary, we confirm that *SLC30A8* LoF is protective against type 2 diabetes, while partial LoF is associated with statistically significant improvements in glucose tolerance and insulin secretion. Our data suggest that complete loss of *SLC30A8* Function is safe and even well tolerated in humans, but is not completely protective against type 2 diabetes. Importantly, *SLC30A8* LoF did not impact BMI (ESM Table 6), and may represent an alternative mechanism for type 2 diabetes treatment and prevention that is independent of currently available weight loss therapies. These results provide strong support for *SLC30A8* knockdown as a safe and effective therapeutic approach for the treatment of type 2 diabetes.

## Supplementary Information

Below is the link to the electronic supplementary material.

ESM (PDF 1.86 MB)

## Data Availability

The whole-exome sequencing data that we have generated includes rare LoF variants, many that have a count of fewer than five, which may potentially lead to identification of study participants. All academic requests to access relevant data should be sent to ks76@cncdpk.com. The Center for Non-Communicable Diseases (CNCD) will ask investigators to sign a data confidentiality agreement that would require investigators to maintain de-identification of the study participants.

## Abbreviations

CIR30: Corrected insulin response
DI: Disposition index
GIP: Gastric inhibitory polypeptide
GLP-1: Glucagon-like peptide-1
ISI: Insulin sensitivity index
LoF: Loss-of-function
PGR: Pakistan Genome Resource
