# Supplementary material for: Complete loss of SLC30A8 in humans improves glucose metabolism and beta cell function
Source: Diabetologia. 2025 Sep 29;68(12):2754–66. doi: 10.1007/s00125-025-06530-3 (PMC12594714; doi:10.1007/s00125-025-06530-3)

ESM Table 1. Summary stats for type 2 diabetes ExWAS. Top hits with at least one gene burden *p*-meta < $5x10^{-7}$ for PGR and UKB Biobank participants. OR, odds ratio; CI, 95% confidence interval; *n*, sample size; MAC, minor allele count; AAF, alternative allele frequency.

		PGR						UK Biobank						Meta-analyzed		
**GeneName: EnsemblGeneId**	**Gene burden**	**OR**	**CI**	***p***	***n***	**MAC**	**AAF**	**OR**	**CI**	***p***	***n***	**MAC**	**AAF**	**OR**	**CI**	***p***
GCK: ENSG00000106633	High confidence pLoFs aaf < 1%	1.00	0.29,3.37	0.99	77778	17	$1x10^{-4}$	17.6	8.8,35	$4.7x10^{-16}$	331754	46	$7x10^{-5}$	8.63	4.9,15	$1.6x10^{-13}$
GCK: ENSG00000106633	High confidence pLoFs + damaging missense aaf < 1%	1.39	0.79,2.44	0.25	119782	63	$3x10^{-4}$	4.07	2.9,5.7	$3.8x10^{-16}$	331754	333	$5x10^{-4}$	2.97	2.3,3.9	$4.2x10^{-15}$
HNF1A: ENSG00000135100	High confidence pLoFs aaf < 1%	1.56	0.53,4.54	0.42	119782	18	$1x10^{-4}$	7.81	4.5,14	$5.6x10^{-13}$	331754	96	$1x10^{-4}$	5.58	3.4,9.2	$1.0x10^{-11}$
HNF1A: ENSG00000135100	High confidence pLoFs + damaging missense aaf < 1%	1.10	0.81,1.51	0.54	119782	172	$7x10^{-4}$	1.65	1.3,2.1	$3.6x10^{-5}$	331754	1056	$2x10^{-3}$	1.43	1.2,1.7	$1.2x10^{-4}$
HNF4A: ENSG00000101076	High confidence pLoFs aaf < 1%	3.35	1.36,8.23	0.008	119782	23	$1x10^{-4}$	5.20	1.8,15	0.003	331754	24	$4x10^{-5}$	3.69	1.9,7.1	$1.1x10^{-4}$
HNF4A: ENSG00000101076	High confidence pLoFs + damaging missense aaf < 1%	1.87	1.24,2.81	0.003	119782	112	$5x10^{-4}$	2.50	1.9,3.4	$1.5x10^{-9}$	331754	523	$8x10^{-4}$	2.25	1.8,2.8	$6.2x10^{-12}$
MAP3K15: ENSG00000180815	High confidence pLoFs aaf < 1%	0.92	0.78,1.08	0.30	119782	437	$2x10^{-3}$	0.77	0.68,0.88	$5.6x10^{-5}$	331754	4018	$6x10^{-3}$	0.84	0.76,0.92	$1.1x10^{-4}$
MAP3K15: ENSG00000180815	High confidence pLoFs + damaging missense aaf < 1%	0.83	0.75,0.92	$2.5x10^{-4}$	119782	1221	$5x10^{-3}$	0.81	0.74,0.88	$2.7x10^{-6}$	331754	7689	$1x10^{-2}$	0.83	0.78,0.88	$1.2x10^{-9}$
PAM: ENSG00000145730	High confidence pLoFs aaf < 1%	0.62	0.24,1.62	0.33	119782	19	$1x10^{-4}$	1.02	0.59,1.8	0.94	331754	260	$4x10^{-4}$	0.93	0.59,1.5	0.77
PAM: ENSG00000145730	High confidence pLoFs + damaging missense aaf < 1%	0.73	0.50,1.06	0.10	119782	126	$5x10^{-4}$	1.29	1.2,1.4	$1.0x10^{-8}$	331754	8846	$1x10^{-2}$	1.25	1.2,1.4	$2.0x10^{-7}$
SLC30A8: ENSG00000164756	High confidence pLoFs aaf < 1%	0.65	0.54,0.77	$1.1x10^{-6}$	119782	712	$3x10^{-3}$	0.42	0.20,0.87	0.020	331754	238	$4x10^{-4}$	0.64	0.54,0.75	$9.4x10^{-8}$
SLC30A8: ENSG00000164756	High confidence pLoFs + damaging missense aaf < 1%	0.63	0.54,0.75	$6.3x10^{-8}$	119782	918	$4x10^{-3}$	0.61	0.44,0.84	0.002	331754	1013	$2x10^{-3}$	0.63	0.55,0.73	$5.0x10^{-10}$

ESM Table 2. Association of predicted LoF variants in *SLC30A8* with T2D individually or in a gene burden test. The AA count includes one individual who is heterozygous for both Arg138Ter and Gln174Ter, and two individuals who are heterozygous for both Gln174Ter and Tyr284Ter. GRCh38-POS, nucleotide position in GRCh38 reference genome; HGVS, Human Genome Variation Society; AAF, alternate allele frequency; R, reference allele, A, alternate allele; OR, odds ratio; CI, confidence interval.

Chr:GRCh38_POS:Ref:Alt	Variant effect	HGVS	AAF	cases RR:RA:AA	controls RR:RA:AA	OR	CI	*p*
**chr8:117146967:C:T**	Stop gain	Gln29Ter	1.27×10^{-5}	32441:2:0	46382:0:0	5.6	0.31, 103	0.24
**chr8:117146988:C:T**	Stop gain	Gln36Ter	1.98×10^{-5}	9201:0:0	16097:1:0	0.21	0.00, 12	0.45
**chr8:117146996:C:CAG**	Frameshift	Pro42AspfsTer45	1.51×10^{-5}	10639:0:0	22573:1:0	0.21	0.00, 12	0.45
**chr8:117147101:ACT:A**	Frameshift	Cys75PhefsTer35	1.90×10^{-5}	32442:1:0	46380:2:0	0.61	0.06, 5.9	0.67
**chr8:117147138:AT:A**	Frameshift	Ile86MetfsTer23	4.52×10^{-5}	10638:1:0	22572:2:0	0.91	0.08, 10	0.94
**chr8:117153084:C:T**	Stop gain	Arg138Ter	0.00086	44927:55:1	74653:144:2	0.62	0.46, 0.84	0.002
**chr8:117153091:G:T**	Splice donor	c.418+1G>T	1.51×10^{-5}	10639:0:0	22573:1:0	0.146	0.00, 7.4	0.34
**chr8:117157690:G:C**	Splice acceptor	419-1G>C	0.00013	44794:7:0	74259:24:0	0.44	0.21, 0.92	0.03
**chr8:117157711:TC:T**	Frameshift	Ile148SerfsTer7	2.94×10^{-5}	44801:0:0	74276:7:0	0.22	0.04, 1.1	0.07
**chr8:117157792:C:T**	Stop gain	Gln174Ter	0.0015	44875:107:1	74551:243:5	0.73	0.58, 0.92	0.007
**chr8:117157827:AG:A**	Frameshift	Val186TrpfsTer7	1.98×10^{-5}	9200:1:0	16098:0:0	4.68	0.08, 280	0.46
**chr8:117157846:T:C**	Splice donor	c.572+2T>C	9.34×10^{-6}	23241:1:0	30284:0:0	9.0	0.18, 460	0.27
**chr8:117161743:CTG:C**	Frameshift	Val194GlyfsTer4	3.95×10^{-5}	9200:1:0	16097:1:0	0.63	0.04, 11	0.75
**chr8:117163424:G:A**	Splice acceptor	c.724-1G>A	3.05×10^{-5}	21559:0:0	43995:4:0	0.2	0.02, 1.8	0.16
**chr8:117163526:G:GT**	Frameshift	Glu276Ter	7.10×10^{-5}	1719:0:0	5326:1:0	0.32	0.00, 130	0.71
**chr8:117171033:G:C**	Splice acceptor	c.830-1G>C	1.87×10^{-5}	23242:0:0	30283:0:1	0.45	0.06, 3.5	0.45
**chr8:117171056:C:A**	Stop gain	Tyr284Ter	0.00034	44779:22:0	74224:59:0	0.66	0.41, 1.1	0.09
**chr8:117171086:C:CA**	Frameshift	Asp295ArgfsTer42	4.04×10^{-5}	33880:1:0	52853:4:1	0.40	0.10, 1.6	0.19

**All LoF**			**0.003**	**44782:199:2**	**74299:488:12**	**0.66**	**0.56, 0.77**	**$3.0x10^{-7}$**

ESM Table 3. Association of damaging missense variants in *SLC30A8* with T2D individually or in a gene burden test combined with predicted LoF. The "All LoF + damaging missense" gene burden test includes missense variants that led to loss of expression of SLC30A8 *in vitro* and the LoF variants listed in ESM Table 1 and is fitted to an additive model. The AA count includes individuals who are compound heterozygous for LoF in ESM Table 1 and compound heterozygous for Arg165His and Arg138Ter. GRCh38-POS, nucleotide position in GRCh38 reference genome; HGVS, Human Genome Variation Society; AAF, alternate allele frequency; R, reference allele, A, alternate allele; OR, odds ratio; CI, confidence interval.

Chr:GRCh38_POS:Ref:Alt	Variant effect	HGVS	AAF	cases RR:RA:AA	controls RR:RA:AA	OR	CI	*p*
**Damaging missense**								
**chr8:117153072:T:A**	Missense	Phe134Ile	$5x10^{-5}$	44800:1:0	74272:11:0	0.32	0.08, 1.2	0.098
**chr8:117153073:T:G**	Missense	Phe134Cys	$4x10^{-5}$	35599:1:0	58179:6:0	0.38	0.08, 1.9	0.24
**chr8:117157765:C:T**	Missense	Arg165Cys	0.00013	44978:5:0	74773:26:0	0.49	0.22, 1.1	0.084
**chr8:117157766:G:A**	Missense	Arg165His	0.00049	44959:24:0	74709:87:3	0.55	0.37, 0.81	0.003
**chr8:117172652:T:G**	Missense	Cys361Gly	$2x10^{-5}$	9201:0:0	16097:1:0	0.21	0.0, 13	0.36
**All LoF + damaging missense**			**0.0037**	**44751:230:2**	**74167:616:16**	**0.62**	**0.53, 0.72**	**$1.12x10^{-9}$**
**Other missense variants**								
**chr8:117172544:C:T**	Missense	Arg325Trp	0.26	28867:13904:2212	45316:25075:4408	0.92	0.90, 0.94	1.9E-15
**chr8:117153025:T:C**	Missense	Leu118Pro	0.00013	44793:8:0	74260:23:0	0.67	0.31, 1.4	0.30
**chr8:117172652:T:G**	Missense	Cys361Gly	$2x10^{-5}$	9201:0:0	16097:1:0	0.21	0.0, 13	0.36

ESM Table 4: PheWAS of aggregated LoF variants and Arg325Trp variants in *SLC30A8* with glycemia related traits. Effect estimates are provided in units of SD of the raw value. *SLC30A8* loss of function heterozygotes and homozygotes demonstrate gene dosage-dependent decreases in plasma glucose and insulin levels. HbA_{1c} data is limited in this cohort. While the direction of effect is negative as expected for a protective effect, it is not statistically significant in LoF variants. A similar but more moderate gene dosage effect is observed for heterozygotes and homozygotes for the common protective variant Arg325Trp. R, reference allele; A, alternate allele; SD, standard deviation; SE, standard error; CI, confidence interval; mmol/l, millimoles per liter; mmol/mol, millimoles per mol; HbA_{1c}, haemoglobin A_{1C}; pmol/l, picomoles per liter.

Variant	Phenotype	Genotype (RR:RA:AA)	Median (RR)	Median (RA)	Median (AA)	Beta (per SD)	SE	CI	*p*
**LoF**	Glucose (mmol/l)	126236:918:17	6.2 (5.0, 9.2)	5.6 (4.7, 7.5)	5.4 (4.7, 6.1)	-0.2	0.04	-0.28, -0.11	4.1×10^{-8}
	HbA_{1c} (mmol/mol)	25278:164:5	44 (39, 58)	44 (38, 55)	38 (36, 40)	-0.1	0.07	-0.24, 0.05	0.066
	HbA_{1c} (%)	25278:164:5	6.2 (5.7, 7.5)	6.2 (5.6, 7.2)	5.6 (5.4, 5.8)	-0.1	0.07	-0.24, 0.05	0.066
	Insulin (pmol/l)	7755:56:02	106 (56.3, 211)	124 (56.3, 372)	581 (532, 632)	0.5	0.13	0.22, 0.72	0.008
**Arg325Trp**	Glucose (mmol/l)	79091|41015|7065	6.2 (5.0, 9.3)	6.2 (5.0, 9.1)	6.1 (4.9, 8.7)	-0.005	0.006	-0.02, 0.01	1.8×10^{-11}
	HbA_{1c} (mmol/mol)	15444|8503|1500	44 (38, 60)	44 (39, 57)	43 (38, 54)	-0.05	0.01	-0.07, -0.03	3.6×10^{-6}
	HbA_{1c} (%)	15444|8503|1500	6.2 (5.6, 7.6)	6.2 (5.7, 7.4)	6.1 (5.6, 7.1)	-0.05	0.01	-0.07, -0.03	3.6×10^{-6}
	Insulin (pmol/l)	4564|2773|476	103 (55.6, 210)	109 (56.3, 211)	119 (64.6, 241)	0.01	0.02	-0.03, 0.05	0.090

ESM Table 5: Summary statistics comparing association of *SLC30A8* LoF + damaging missense with glycemic traits with and without adjustment for Arg325Trp. Binary trait effect sizes are reported as ln(OR). UCI, upper confidence interval; LCI, lower confidence interval; SE, standard error.

		Not adjusted					Adjusted for Arg325Trp				
**Phenotype**	**Trait type**	**Beta**	**UCI**	**LCI**	**SE**	***p***	**Beta**	**UCI**	**LCI**	**SE**	***p***
Type 2 diabetes	Binary	-0.48	-0.32	-0.63	0.08	1.1×10^{-9}	-0.50	-0.34	-0.65	0.08	3.0×10^{-10}
Family history diabetes	Binary	-0.17	-0.02	-0.32	0.08	0.022	-0.18	-0.03	-0.33	0.08	0.017
Glucose	Continuous	-0.23	-0.15	-0.31	0.04	4.1×10^{-8}	-0.24	-0.16	-0.32	0.04	1.2×10^{-8}
HbA_{1c}	Continuous	-0.13	0.01	-0.27	0.07	0.066	-0.14	-0.001	-0.28	0.07	0.049
Insulin	Continuous	0.33	0.57	0.09	0.12	0.008	0.33	0.58	0.09	0.12	0.007

ESM Table 6. Baseline characteristics and binary traits of *SLC30A8* LoF and damaging missense carriers by genotype. Reported *p* values were generated by adjusting for age, age^2, sex, age x sex, top 10 genetic principal components and whole genome regression predictions. ALT, alanine aminotransferase; AST, aspartate aminotransferase; ASCVD, atherosclerotic cardiovascular disease, BP, blood pressure; BPM, beats per minute; BMI, body mass index; eGFR, estimated glomerular filtration rate; FGF23, fibroblast growth factor 23; GGT, gamma-glutamyl transferase; HDL-C, high-density lipoprotein cholesterol; HbA_{1c}, haemoglobin A_{1c}; kg, kilograms; l, liter; LDL, low-density lipoprotein cholesterol; m, meters; μmol, micromoles; mmol, millimoles; mg, milligrams; nmol, nanomoles; pmol, picomoles U, units; WHR, waist-to-hip ratio. All analyses were performed using linear mixed models with age and gender as fixed effects, and family ID as a random effect. Carrier numbers are shown as HomRR:HetRA:HomAA.

Characteristic	Genotype counts	Non-carriers	Heterozygotes	Knockouts	*p* additive	*p* recessive
Female	143885:1023:18	52518 (36%)	396 (39%)	3 (17%)		
Age	143885:1023:18	50 (42, 60)	50.0 (40, 60)	55 (57, 75)		
BMI (kg/m^2)	97779:689:14	26.6 (23.9, 29.8)	26.9 (24.2, 30.1)	27.3 (25.6, 30.0)	0.33	0.62
Weight (kg)	102549:735:14	70 (64.0, 80.0)	72 (64.0, 80.0)	75.5 (70.5, 79.5)	0.15	0.50
Height (m)	98717:695:15	163 (157.0, 169.0)	163 (157.0, 169.0)	168 (163.0, 170.0)	0.13	0.90
WHR	95303:672:15	0.958 (0.92, 0.98)	0.959 (0.93, 0.99)	0.931 (0.9, 0.97)	0.54	0.55
Heart rate (BPM)	28403:199:4	78 (70.0, 88.0)	80 (73.0, 90.0)	83 (81.5, 88.0)	0.034	0.22
BP diastolic	120635:863:17	80 (80.0, 90.0)	80 (79.0, 90.0)	80 (80.0, 80.0)	0.59	0.74
BP systolic	121811:867:17	128 (120.0, 140.0)	120 (120.0, 140.0)	120 (120.0, 130.0)	0.35	0.95
Glucose (mmol/l)	126236:918:17	6.2 (5.0, 9.2)	5.6 (4.7, 7.5)	5.4 (4.7, 6.1)	4.1×10^{-8}	0.11
HbA_{1c} (mmol/mol)	25278:164:5	44.3 (37.7, 58.5)	44.3 (37.7, 55.2)	37.7 (35.5, 39.9)	0.066	0.086
HbA_{1c} (%)	25278:164:5	6.2 (5.6, 7.5)	6.2 (5.6, 7.2)	5.6 (5.4, 5.8)	0.066	0.086
Insulin (pmol/l)	7755:56:02	91.2 (48.5, 182)	107 (48.6, 322)	502 (460, 546)	0.008	0.021
Creatinine (µmol/l)	119730:858:15	72.5 (59.2, 88.4)	72.5 (56.6, 88.4)	70.7 (63.6, 86.6)	0.42	0.041
eGFR	114388:824:14	90.8 (73.7, 111)	91.0 (73.9, 111)	89.1 (77.0, 106)	0.61	0.070
Cystatin C (mg/l)	21430:146:2	1.0 (0.80, 1.2)	1.1 (0.80, 1.3)	1.2 (0.98, 1.3)	0.031	0.66
C peptide (nmol/l)	8919:58:2	0.33 (0.21, 0.52)	0.39 (0.19, 0.56)	0.81 (0.74, 0.88)	0.14	0.038
Total cholesterol (mmol/l)	139423:998:18	4.5 (3.7, 5.3)	4.4 (3.7, 5.2)	4.2 (3.1, 4.7)	0.72	0.090
HDL (mmol/l)	139105:995:17	0.91 (0.75, 1.1)	0.91 (0.75, 1.1)	0.83 (0.70, 0.93)	0.21	0.70
Triglycerides (mmol/l)	139119:997:18	1.8 (1.3, 2.7)	1.7 (1.2, 2.5)	1.8 (1.5, 3.3)	0.0054	0.45
LDL (mmol/l)	129259:941:12	2.5 (1.9, 3.2)	2.5 (1.9, 3.2)	2.7 (1.9, 3.1)	0.53	0.87
ALT (U/l)	53554:410:10	19.5 (13.0, 30.0)	19.8 (12.9, 29.7)	21.0 (16.6, 37.8)	0.97	0.83
AST (U/l)	54018:413:10	21.4 (16.5, 28.7)	21.4 (16.8, 29.3)	29.5 (21.2, 36.2)	0.20	0.08
GGT (U/l)	21454:146:2	22.0 (15.0, 36.0)	22.0 (15.0, 37.0)	60.0 (59.5, 60.5)	0.59	0.097
Uric acid (µmol/l)	21198:145:2	297 (232, 357)	309 (250, 387)	419 (394, 445)	0.003	0.078
FGF23	8375:60:2	69 (49, 105)	68 (49, 88)	58 (53, 63)	0.52	0.75
Type 2 diabetes	44751:230:2 cases<br>74167:616:16 controls	44751<br>(38%)	230<br>(27%)	2<br>(11%)	1.1×10^{-9}	0.038
Angina	4088:33:1 cases<br>87310:642:12 controls	4088<br>(5%)	33<br>(5%)	1<br>(8%)	0.002	0.60
Myocardial infarction	33803:237:2 cases<br>75554:558:11 controls	33803<br>(40%)	237<br>(31%)	2<br>(15%)	0.18	0.054
ASCVD	44687:295:5 cases<br>87433:640:12 controls	44687<br>(43%)	295<br>(34%)	5<br>(29%)	0.30	0.43

Hypertension	50684:376:6 cases<br>91705:641:12 controls	50684<br>(37%)	376<br>(36%)	6<br>(33%)	0.56	0.90
Family history diabetes	33682:198:3 cases<br>110298:826:15 controls	33682<br>(20%)	198<br>(23%)	3<br>(17%)	0.022	0.78
Family history myocardial infarction	18087:111:2 cases<br>125893:913:16 controls	18087<br>(14%)	111<br>(13%)	2<br>(11%)	0.14	0.62

ESM Figure 1. Conservation analysis of SLC30A8. Three nonsense (red boxes) and seven missense (yellow boxes) variants, including Arg325Trp, were selected for *in vitro* expression profiling based on (A) conservation analysis cross *SLC30A8* orthologs or (B) disruption of critical structural features in SLC30A8. (C) cDNA constructs of SLC30A8 variants were expressed in HEK293 cell lysates and detected by western blot against an N-terminal FLAG tag as described previously[25] to allow detection of misfolded forms of protein which abolish epitopes for SLC30A8-specific antibodies. Dashed line indicates where image has been cropped to splice together empty lanes. Monomeric wild-type SLC30A8 (Lane 1) runs as a 30kDa band. SDS-resistant multimers run at 50kDa or higher. Relative band intensities normalized to housekeeping protein are displayed. Variants that resulted in loss of expression or significant aggregation relative to wild-type were classified as loss-of-function and included in ExWAS and PheWAS analysis as aggregated “LoF+ damaging missense.” Western blots are representative images from three biological replicates.

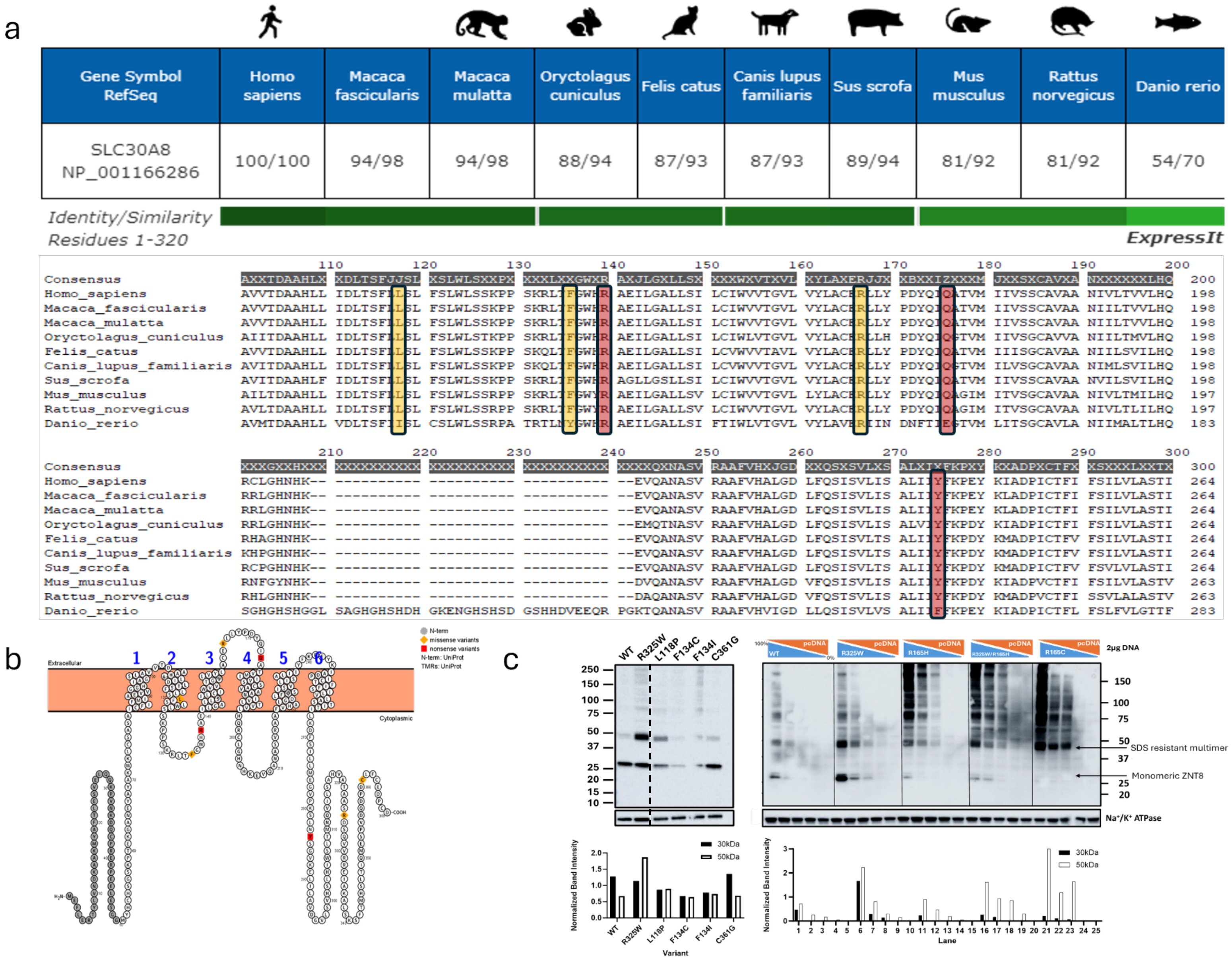
a
Gene Symbol RefSeq
Homo sapiens
Macaca fascicularis
Macaca mulatta
Oryctolagus cuniculus
Felis catus
Canis lupus familiaris
Sus scrofa
Mus musculus
Rattus norvegicus
Danio rerio
SLC30A8 NP_001166286
100/100
94/98
94/98
88/94
87/93
87/93
89/94
81/92
81/92
54/70
Identity/Similarity
Residues 1-320
ExpressIt
Consensus
Homo_sapiens
Macaca_fascicularis
Macaca_mulatta
Oryctolagus_cuniculus
Felis_catus
Canis_lupus_familiaris
Sus_scrofa
Mus_musculus
Rattus_norvegicus
Danio_rerio
b
Extracellular
Cytoplasmic
N-term
missense variants
nonsense variants
N-term: UniProt
TMRs: UniProt
c
WT
R325W
L118P
F134C
F134I
C361G
2μg DNA
pcDNA
R165H
R325W/R165H
R165C
SDS resistant multimer
Monomeric ZNT8
Na⁺/K⁺ ATPase
Normalized Band Intensity
30kDa
50kDa
Variant
Lane

ESM Figure 2. Analysis of SLC30A8 Arg325Trp variant OGTTs. (**a**) To test effects of common protective variant Arg325Trp, OGTT results from non-diabetic recall participants were separated by Arg325Trp genotype and re-analyzed. Reference (*n*=121, blue) do not carry LoF or Arg325Trp alleles; Heterozygous (*n*=135, green) and Homozygous (*n*=33, red) Arg325Trp carry one and two Arg325Trp alleles, respectively. (*) denotes statistical significance (p<0.05). (**b**) Corrected insulin response (CIR30) and Matsuda Insulin Sensitivity Index calculated for each group from fasting insulin and glucose. Center line, median; box limits, upper and lower quartiles; whiskers, farthest data point within 1.5x interquartile range; all points displayed.

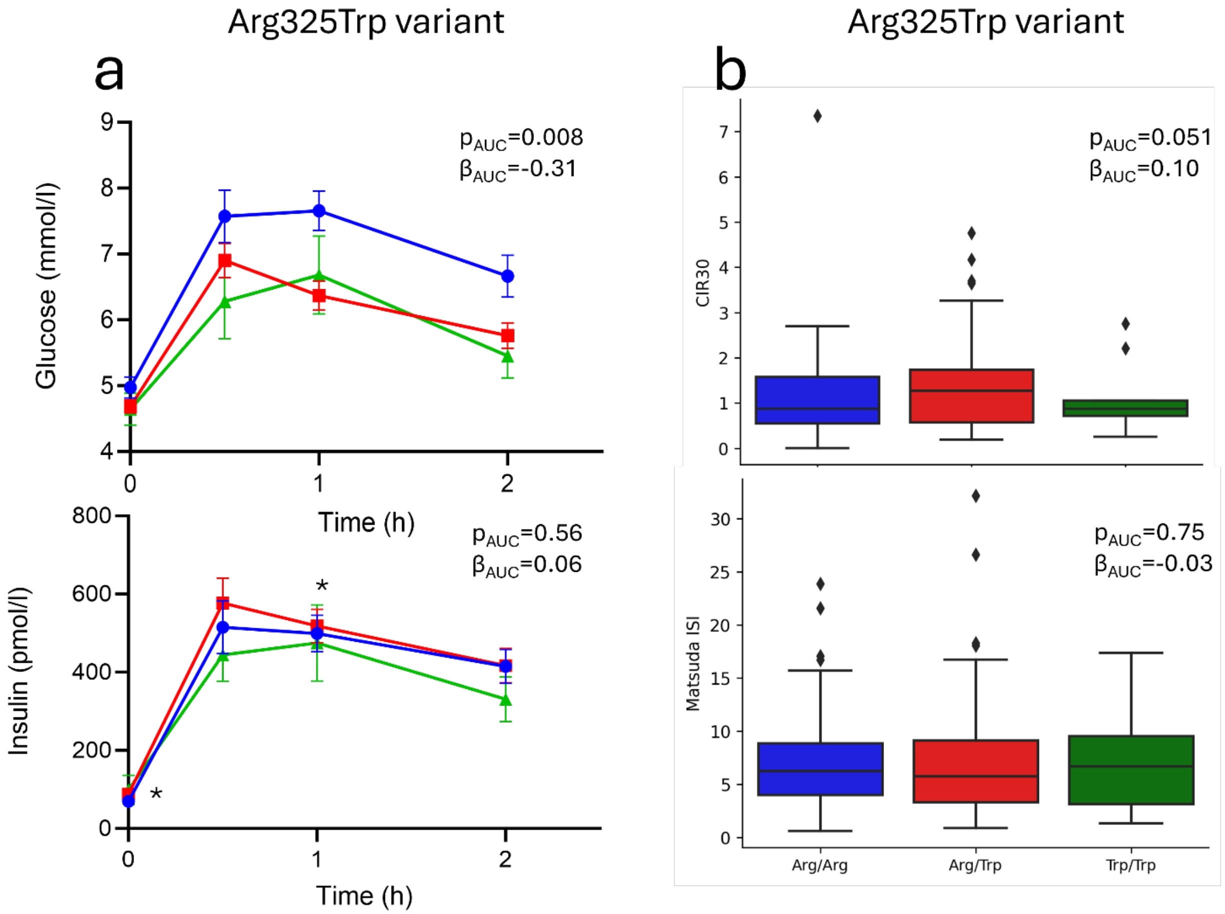

ESM Figure 3. Insulin measurements in Arg325Trp or LoF carriers. Insulin processing was analyzed with the ratios of proinsulin to insulin and proinsulin to C-peptide in OGTT samples. Similar patterns emerged across LoF and Arg325Trp heterozygotes and homozygotes in which insulin processing efficiency tended to increase slightly in a gene-dose dependent manner. **p*<0.05, ***p*<0.01, ****p*<0.001

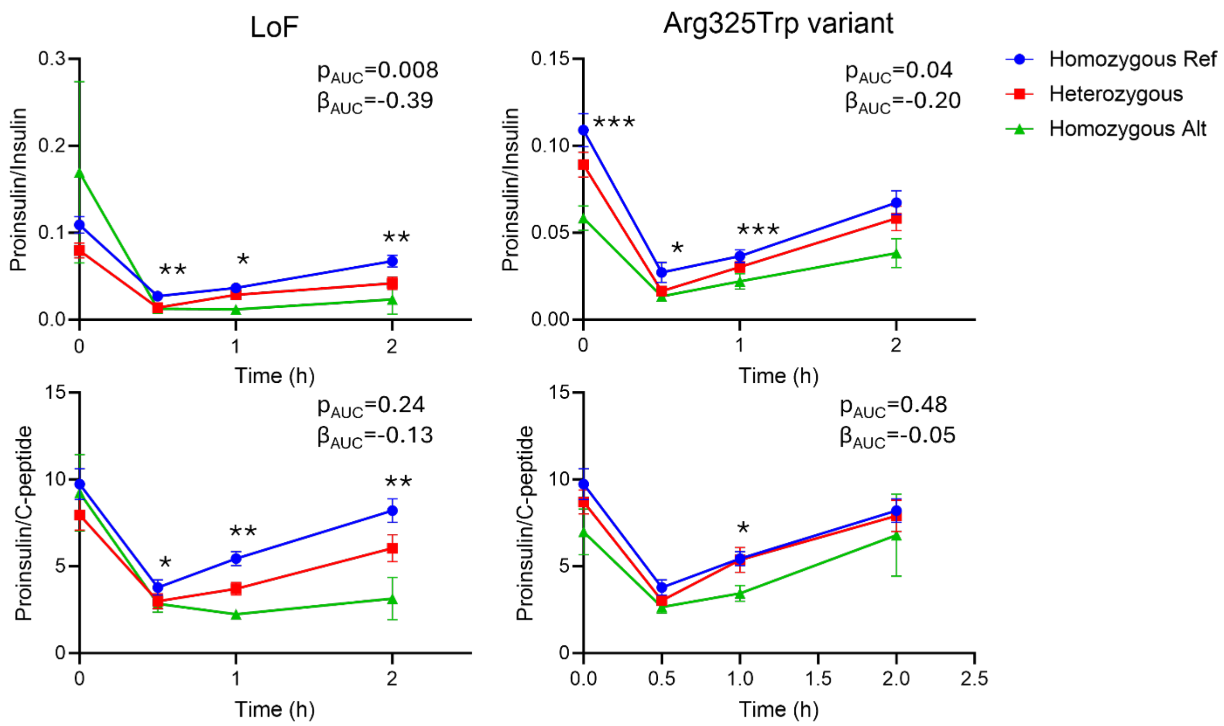

Supplement: Supplementary file 1 — ESM (PDF 1.86 MB) [file 125_2025_6530_MOESM1_ESM.pdf]
